# Efficacy evaluation of three-dimensional printing assisted osteotomy guide plate in accurate osteotomy of adolescent cubitus varus deformity

**DOI:** 10.1186/s13018-019-1403-7

**Published:** 2019-11-09

**Authors:** Yuan-Wei Zhang, Xin Xiao, Wen-Cheng Gao, Yan Xiao, Su-Li Zhang, Wen-Yan Ni, Liang Deng

**Affiliations:** 1 Department of Orthopedics, Jiangxi Provincial People’s Hospital Affiliated to Nanchang University, No.152 Aiguo Road, Nanchang, 330006 Jiangxi China; 2 Medical Department of Graduate School, Nanchang University, Nanchang, 330006 Jiangxi China; 3 Department of Orthopedics, Dongguan Houjie Town People’s Hospital, Dongguan, 523900 Guangdong China; 4 Department of Operating room, Wujin Hospital Affiliated to Jiangsu University, Changzhou, 213161 Jiangsu China

**Keywords:** Cubitus varus deformity, Three-dimensional printing, Osteotomy guide plate, Humeral supracondylar wedge osteotomy

## Abstract

**Background:**

This present study is aimed to retrospectively assess the efficacy of three-dimensional (3D) printing assisted osteotomy guide plate in accurate osteotomy of adolescent cubitus varus deformity.

**Material and methods:**

Twenty-five patients (15 males and 10 females) with the cubitus varus deformity from June 2014 to December 2017 were included in this study and were enrolled into the conventional group (*n* = 11) and 3D printing group (*n* = 14) according to the different surgical approaches. The operation time, intraoperative blood loss, osteotomy degrees, osteotomy end union time, and postoperative complications between the two groups were observed and recorded.

**Results:**

Compared with the conventional group, the 3D printing group has the advantages of shorter operation time, less intraoperative blood loss, higher rate of excellent correction, and higher rate of the parents’ excellent satisfaction with appearance after deformity correction (*P* < 0.001, *P* < 0.001, *P* = 0.019, *P* = 0.023). Nevertheless, no significant difference was presented in postoperative carrying angle of the deformed side and total complication rate between the two groups (*P* = 0.626, *P* = 0.371).

**Conclusions:**

The operation assisted by 3D printing osteotomy guide plate to correct the adolescent cubitus varus deformity is feasible and effective, which might be an optional approach to promote the accurate osteotomy and optimize the efficacy.

## Background

Distal humeral fracture is the most usual upper limb fracture in adolescents, with an incidence of about 60% in all elbow fractures [1]. Cubitus varus deformity, as the most common complication of the distal humeral fractures in adolescents, accounts for approximately 30% to 58% [2, 3]. In addition to the varus on the coronal plane, the cubitus varus can also include the three-plane deformities of the overextension on the sagittal plane and the internal rotation on the horizontal plane [4]. Besides, due to the poor ability of distal humeral epiphysis to correct the existed varus deformity, the cubitus varus deformity will persist into adulthood without any improvement [5, 6]. Thus, the cubitus varus deformity will not only affect the esthetic appearance and restrict the elbow motion, but also delay the daily life and learning of adolescents to a certain extent [7].

Since the humeral supracondylar wedge osteotomy (HSWO) was first proposed in 1939 [8], it has become the most significant surgical procedure to correct the cubitus varus deformity [9, 10]. However, the focus of the operation is often to correct the varus on the coronal plane, which neglects the sagittal or horizontal deformities and results in the unsatisfactory correction effects [11, 12]. Although partial surgeons subjectively hope to implement the three-dimensional correction [11], however, due to the varied difference of deformities in individuals, it is difficult to obtain the truly precise correction under the small incision during the operation, which requires repeated attempts, with long operation time and a large amount of blood loss. In order to improve the preciseness, the HSWO requires to be more accurate and individualized. With the development of digital medicine, especially the rapid advancement of 3D printing technology currently, personalized guide plates can be designed to assist the precise implementation of orthopedic operations [13, 14].

Therefore, to further assess the efficacy of 3D printing assisted osteotomy guide plate in accurate osteotomy of adolescent cubitus varus deformity, this present study retrospectively compared the conventional surgery with surgery assisted by 3D printing technology and assessed the prognosis.

## Material and methods

### Patients

A total of 25 patients (15 males and 10 females) with cubitus varus deformity who were admitted to the Jiangxi Provincial People’s Hospital Affiliated with Nanchang University from June 2014 to December 2017 were included in this study. Patients were enrolled into the conventional group (*n* = 11) and 3D printing group (*n* = 14) by the random number table method after admission and treated with the different surgical procedures, respectively. The cubitus varus deformity of all patients was caused by the malunion of humeral supracondylar fracture, and there was no bone metabolic disease. This study was approved by the Ethics Review Committee of Jiangxi People’s Hospital Affiliated to Nanchang University, and all the patients signed an informed consent to participate in the study.

### Osteotomy angle design and osteotomy guide plate fabrication

**Fig. 1:**
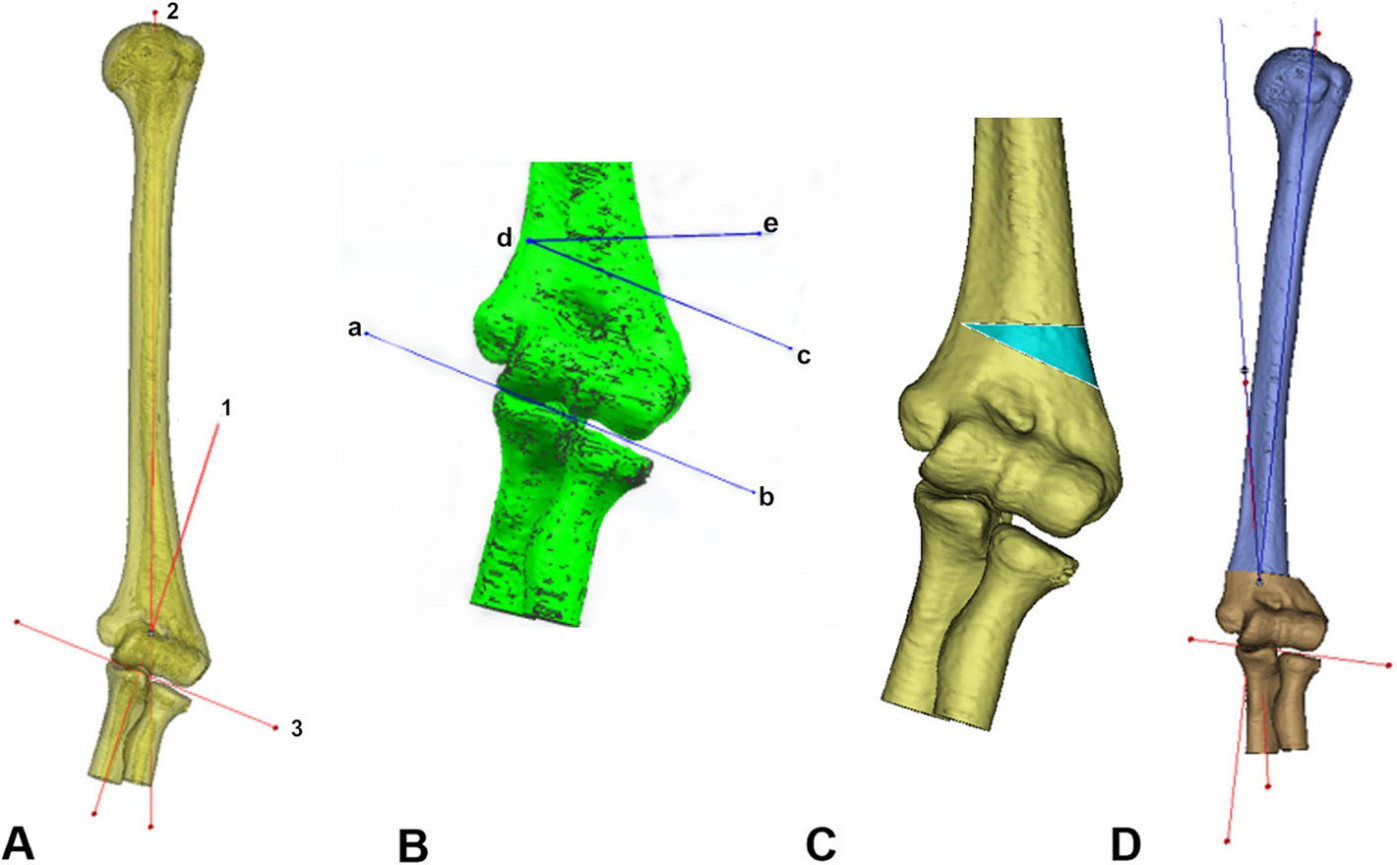
Design of osteotomy angle and planes. **a** Measurements of carrying angle and cubitus varus angle. Line 1, longitudinal axis of the ulna; line 2, longitudinal axis of the humerus; line 3, the elbow joint line. **b** Determination of osteotomy angle. Line ab, the elbow joint line; line cd, parallel line of elbow joint line. **c** Simulation of osteotomy angle. **d** Simulated reduction of bone ends after osteotomy

**Fig. 2:**
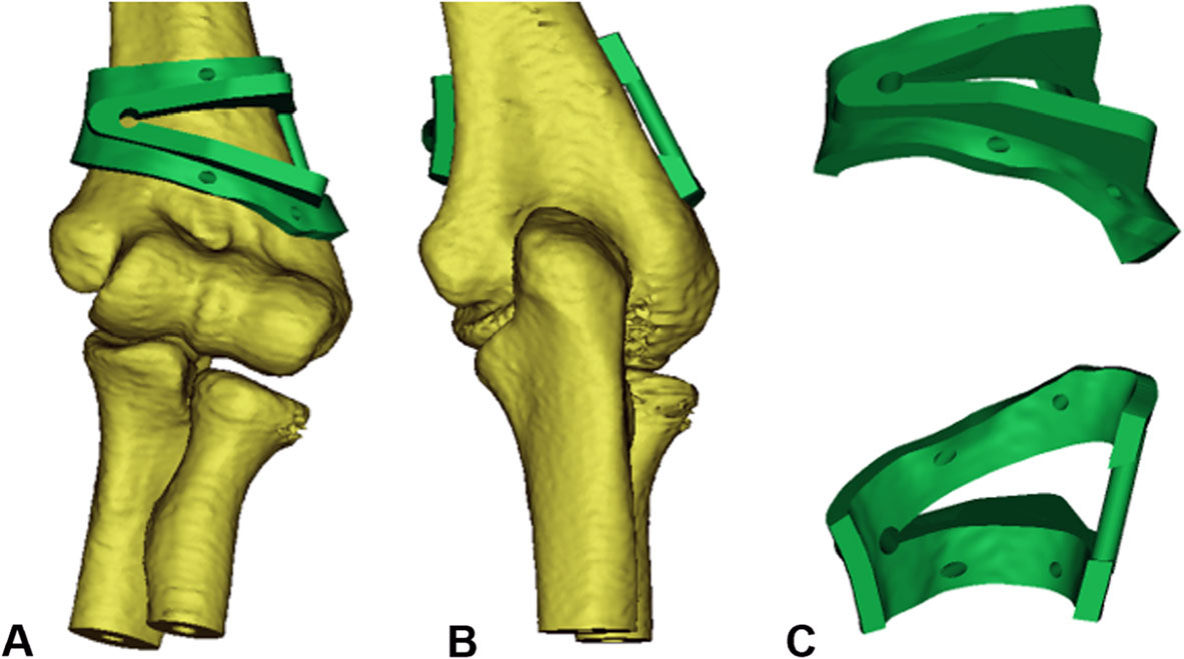
Design of osteotomy guide plate. **a** Positive view. **b** Back view. **c** Front and back of the guide plate

All patients were photographed with the X-rays and computed tomography (CT) of bilateral upper limbs, and the carrying angle of the healthy side and the cubitus varus angle of the deformed side were measured, respectively. On the basis of the previous studies by Takeyasu et al. [15], the osteotomy angle was calculated to be the sum of the carrying angle of the healthy side and the cubitus varus angle of the deformed side. In addition, the CT scanning data of patients in 3D printing group were collected by the dual-source 64-slice spiral CT system (Siemens, Germany) in our hospital, and the scanning parameters were voltage 120 KV and pitch 0.625 mm. The initial CT data of deformed upper limbs were stored in Digital Imaging and Communications in Medicine (DICOM) format and input into the Mimics 19.0 software (Materialize, Leuven, Belgium) to generate three-dimensional reconstructed upper limb models. Then, the osteotomy plane was defined parallel to the articular surface 1 cm above the olecranon, and the other osteotomy plane was defined by the calculated osteotomy angle. The two planes intersected at 2 mm from the medial bone cortex. The simulated reduction of bone ends after osteotomy was then performed, and the carrying angle after osteotomy was measured again to check whether the cubitus varus deformity was corrected (Fig. 1). Then, the anatomical data corresponding to the bony surface of distal humerus were extracted and treated with reverse thickening of 5 mm. The basal plate with the same shape was established, and the data of Kirschner wire guide holes were imported at the same time. After Boolean operation, all guide holes were penetrated and the boundary was trimmed to complete the design and fabrication of the guide plate (Fig. 2). Ultimately, the design data were saved in STL format and output to 3D printer (Dongwang Med, Inc., Xian, Shanxi, China), and the osteotomy guide plate was then printed with materials of photosensitive resin.

### Surgical procedures

**Fig. 3:**
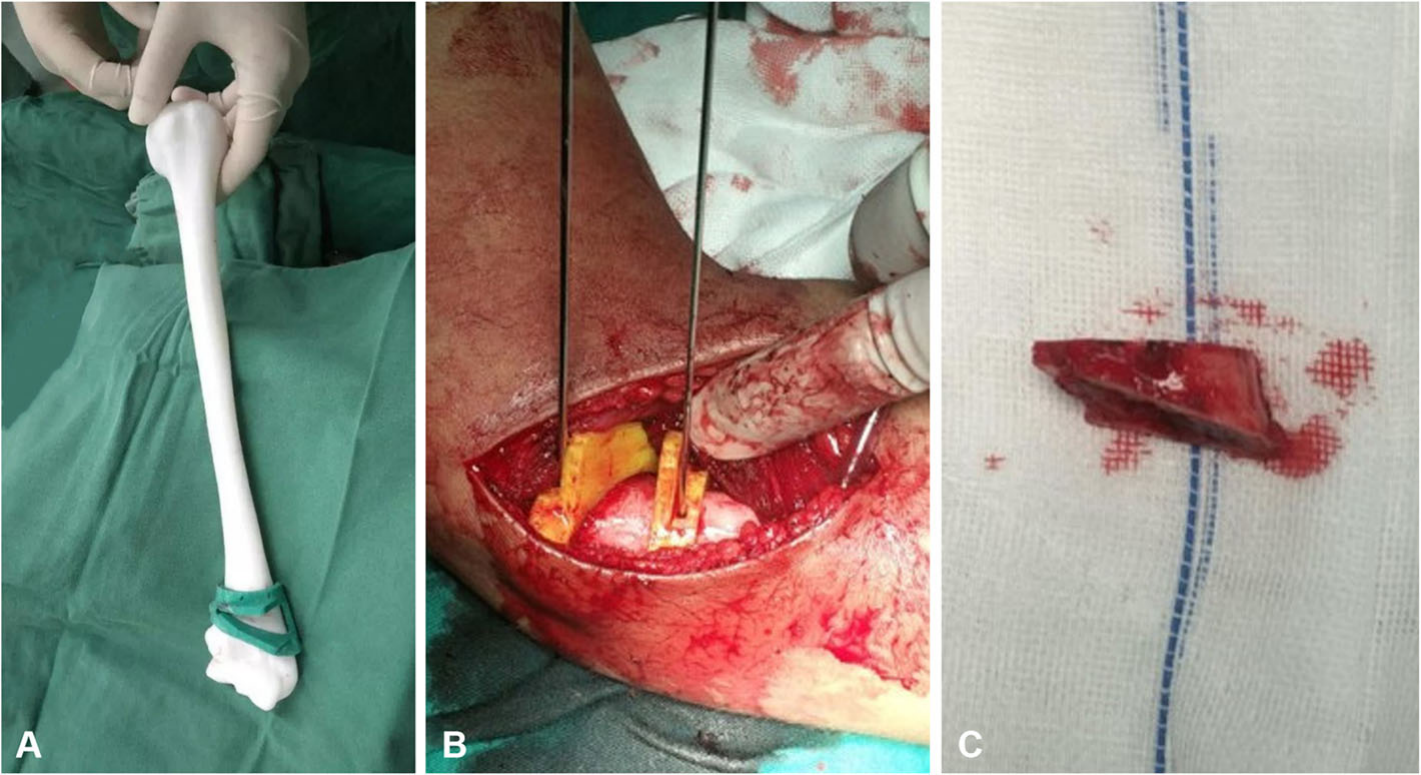
Simulated and intraoperative operation. **a** Simulated operation. **b** The osteotomy guide plate is firmly attached to the distal humerus. **c** The wedge-shaped osteotomy block

All operative procedures were performed by senior surgeons in the same treatment team. Under the general anesthesia or brachial plexus block anesthesia, the patient was placed into a supine position and a pneumatic tourniquet was used to block the blood circulation at the root of the upper limb. In 3D printing group, the placement of osteotomy guide plate and the determination of osteotomy lines were directly referred to the results of preoperative simulation. In this process, two Kirschner wires with a diameter of 2.0 mm were drilled into the humeral shaft along the guide plate holes to fix the osteotomy guide plate. After the firm fixation, the wedge-shaped osteotomy was performed along the upper and lower osteotomy planes of the guide plate, and the medial bone cortex was preserved (Fig. 3). However, the determination of osteotomy angle and osteotomy lines in the conventional group were only based on the preoperative measurements and intraoperative attempts. After checking the carrying angle was satisfactory, different specifications of Kirschner wires or reconstruction locking plates (Dabo Med, Inc., Xiamen, Fujian, China) were selected for shaping and fixation, and the incision was routinely closed with 2/0 absorbable sutures.

### Postoperative management

There was no significant difference in postoperative management between the two groups. Patients in the two groups were treated with long arm plaster support to fix the upper limb. Moreover, postoperative radiographs of the elbow joint were reexamined regularly, and the healing of the incision, range of elbow joint motion, carrying angle, postoperative complications, and the healing of osteotomy ends were observed and recorded. Until the imaging examinations confirmed the formation of continuous callus at the osteotomy end, the plaster support was removed and the patient was guided to start partial weight-bearing exercise and gradually transitioned to complete weight-bearing.

### Parameters evaluation

The parameters of operation time, intraoperative blood loss, osteotomy degrees, and osteotomy end union time of patients between two groups were observed and recorded. Moreover, at the last follow-up, the prognosis of cubitus varus deformity was evaluated based on the Bellemore criteria [16]: (1) excellent: the difference of carrying angle between the deformed side and healthy side was less than 5°, and the restriction of elbow joint flexion and extension was less than 10°; (2) good: the difference of carrying angle between the deformed side and healthy side was 6° to 10°, and the restriction of elbow joint flexion and extension was 11° to 15°; (3) fair: the difference of the carrying angle between the deformed side and healthy side was 11° to 15°, and the restriction of elbow joint flexion and extension was 15° to 20°; and (4) poor: the restriction of elbow joint flexion and extension was more than 20°, accompanied with residual varus deformity, or the complications requiring secondary surgery. In addition, parents’ satisfaction with appearance after the deformity correction was also recorded. In the scoring mechanism with a full score of 100 points, a score ≥ 90 was considered as excellent, 75–89 as good, 50–74 as fair, and < 50 as poor.

### Statistical analysis

The overall data in this study were statistically analyzed by the SPSS 23.0 software (SPSS, Inc., Chicago, USA) and manifested as count (percentage) or mean ± standard deviation (SD). Student’s *t* test, chi-squared test, and Fisher’s exact test were applied to analyze the data in this study. Different parameters measured between two groups were evaluated with independent *t* test for continuous variables, and chi-square test or Fisher’s exact test for the categorical variables. A *P* value < 0.05 was regarded as statistically significant.

## Results

### Demographic data and deformity characteristics

Table 1 revealed the demographic data and deformity characteristics of patients in two groups. Therein, the carrying angle of the healthy side in the conventional group and 3D printing group was 9.6 ± 1.3° and 10.1 ± 1.4° respectively, and the carrying angle of the deformed side was − 17.8 ± 6.3° and − 18.3 ± 6.7°, respectively. However, the comparison between two groups showed no statistical significance in gender, age, causes of deformity, time from fracture to operation, deformity side, and preoperative carrying angle of healthy and deformed sides (*P* > 0.05 for all).

**Table 1:** Comparison of demographic data and deformity characteristics between two groups of adolescents

Characteristics	Conventional group (*n* = 11)	3D printing group (*n* = 14)	*P* value
Mean age (range), years	9.6 ± 3.4 (6–17)	9.9 ± 3.6 (5–18)	0.753
Time from fracture to operation (range), months	25.8 ± 10.2 (12–44)	26.4 ± 9.4 (10–43)	0.376
Gender, *n* (%)			0.577
Male	7 (63.6)	8 (57.1)	
Female	4 (36.4)	6 (42.9)	
Deformity side, *n* (%)			0.191
Left	6 (54.6)	6 (42.9)	
Right	5 (45.4)	8 (57.1)	
Causes of deformity, *n* (%)			0.623
Supracondylar fracture of humerus	11 (100)	14 (100)	
Internal condyle fracture of the humerus	0	0	
Epiphysis injury of the internal condyle	0	0	
Carrying angle of healthy side, °	9.6 ± 1.3	10.1 ± 1.4	0.167
Carrying angle of deformed side, °	− 17.8 ± 6.3	− 18.3 ± 6.7	0.269

### Clinical data

Table 2 revealed the clinical data of patients in two groups. There was statistical significance in operation time between the conventional group (73.5 ± 10.3 min) and 3D printing group (48.3 ± 8.9 min, *P* < 0.001). The intraoperative blood loss in the 3D printing group (35.6 ± 9.7 ml) was significantly less than that of the conventional group (52.1 ± 11.5 ml, *P* < 0.001). Regarding the osteotomy degrees, there was no statistical significance between the conventional group (24.9 ± 6.6°) and 3D printing group (25.7 ± 7.1°, *P* = 0.183). As for the osteotomy ends union time, there was also no statistical significance between the conventional group (9.8 ± 2.7 weeks) and 3D printing group (9.3 ± 2.1 weeks, *P* = 0.417). According to the Bellemore criteria [16], 12 patients in the 3D printing group obtained excellent correction, 1 patient obtained good prognosis, and 1 patient obtained fair prognosis, while 8 patients obtained excellent correction, 2 patients obtained good prognosis, and 1 patient obtained fair prognosis in the conventional group. In addition, it is worth noting that the rate of excellent deformity correction in the 3D printing group (85.8%) was significantly higher than that of the conventional group (72.7%, *P* = 0.019).

**Table 2:** Comparison of clinical data between two groups of adolescents

Clinical data	Conventional group (*n* = 11)	3D printing group (*n* = 14)	*P* value
Operation time, min	73.5 ± 10.3	48.3 ± 8.9	< 0.001
Intraoperative blood loss, ml	52.1 ± 11.5	35.6 ± 9.7	< 0.001
Osteotomy degrees, °	24.9 ± 6.6	25.7 ± 7.1	0.183
Osteotomy ends union time, week	9.8 ± 2.7	9.3 ± 2.1	0.417
Deformity correction, *n* (%)			
Excellent	8 (72.7)	12 (85.8)	0.019
Good	2 (18.2)	1 (7.1)	0.193^a^
Fair	1 (9.1)	1 (7.1)	0.231^a^
Poor	0	0	–
Rate of excellent deformity correction, %	72.7	85.8	0.019

^a^*P* value for continuity-corrected chi-squared test

### Postoperative functional outcomes

Table 3 revealed the postoperative functional outcomes of patients in two groups. There was no statistical significance in the follow-up time between the conventional group (17.8 ± 3.1 months) and 3D printing group (18.3 ± 2.9 months, *P* = 0.317). The carrying angle of the deformed side after correction in the conventional group and 3D printing group was 8.3 ± 2.6° and 8.7 ± 2.4°, respectively, which is not statistically different (*P* = 0.626). Furthermore, the motion of flexion was 128.7 ± 4.9° in the conventional group and for the 3D printing group was 131.6 ± 5.8° (*P* = 0.451). The motion of rotation was 143.5 ± 7.7° in the conventional group and for the 3D printing group was 146.2 ± 8.1° (*P* = 0.192). The motion of extension was 2.4 ± 1.1° in the conventional group and for the 3D printing group was 2.6 ± 0.9° (*P* = 0.523). Besides, no significant difference in the range of elbow joint motions was noted between the two groups at the last follow-up. Regarding the parents’ satisfaction with appearance after deformity correction, nine patients in the conventional group were regarded as excellent, and two patients were regarded as good. However, 13 patients in the 3D printing group were regarded as excellent, and one patient was regarded as good. Compared with the conventional group (81.8%), the 3D printing group (92.9%, *P* = 0.023) exhibited a higher rate of excellent satisfaction.

**Table 3:** Comparison of postoperative functional outcomes between two groups of adolescents

Outcomes	Conventional group (*n* = 11)	3D printing group (*n* = 14)	*P* value
Follow-up time, month	17.8 ± 3.1	18.3 ± 2.9	0.317
Postoperative carrying angle of deformed side, °	8.3 ± 2.6	8.7 ± 2.4	0.626
Range of elbow joint motion at last follow-up, °			
Flexion	128.7 ± 4.9	131.6 ± 5.8	0.451
Rotation	143.5 ± 7.7	146.2 ± 8.1	0.192
Extension	2.4 ± 1.1	2.6 ± 0.9	0.523
Parents’ satisfaction with appearance after deformity correction, *n* (%)			
Excellent	9 (81.8)	13 (92.9)	0.022
Good	2 (18.2)	1 (7.1)	0.625^a^
Fair	0	0	–
Poor	0	0	–
Rate of excellent satisfaction, %	81.8	92.9	0.023

^a^*P* value for continuity-corrected chi-squared test

### Complications

Table 4 revealed the postoperative complications of patients in two groups. Therein, one patient in the conventional group developed the ulnar nerve paralysis after operation, and the symptoms disappeared after 3 weeks of treatment with neurotrophic drugs. Besides, two patients in the 3D printing group and one patient in the conventional group have experienced the temporary restriction of elbow joint motion, but returned to normal after the removal of plaster support and the performance of progressive functional exercise. In addition, no other complications such as the incision infection, osteotomy nonunion, and internal fixation loose or broken were observed in the remaining patients. The total complication rate of the conventional group and 3D printing group was 2 out of 11 (18.2%) and 2 out of 14 (14.3%), with no statistical significance (*P* = 0.371).

**Table 4:** Comparison of complications between two groups of adolescents

Complications	Conventional group (*n* = 11)	3D printing group (*n* = 14)	*P* value
Incision infection	0	0	–
Nerve injury	1 (9.1)	0	1.000^a^
Osteotomy nonunion	0	0	–
Limited elbow motion	1 (9.1)	2 (14.3)	1.000^a^
Internal fixation loose or broken	0	0	–
Total	2 (18.2)	2 (14.3)	0.371

Values are expressed as *n* (%)

^a^*P* value for Fisher’s exact test

### Typical case

**Fig. 4:**
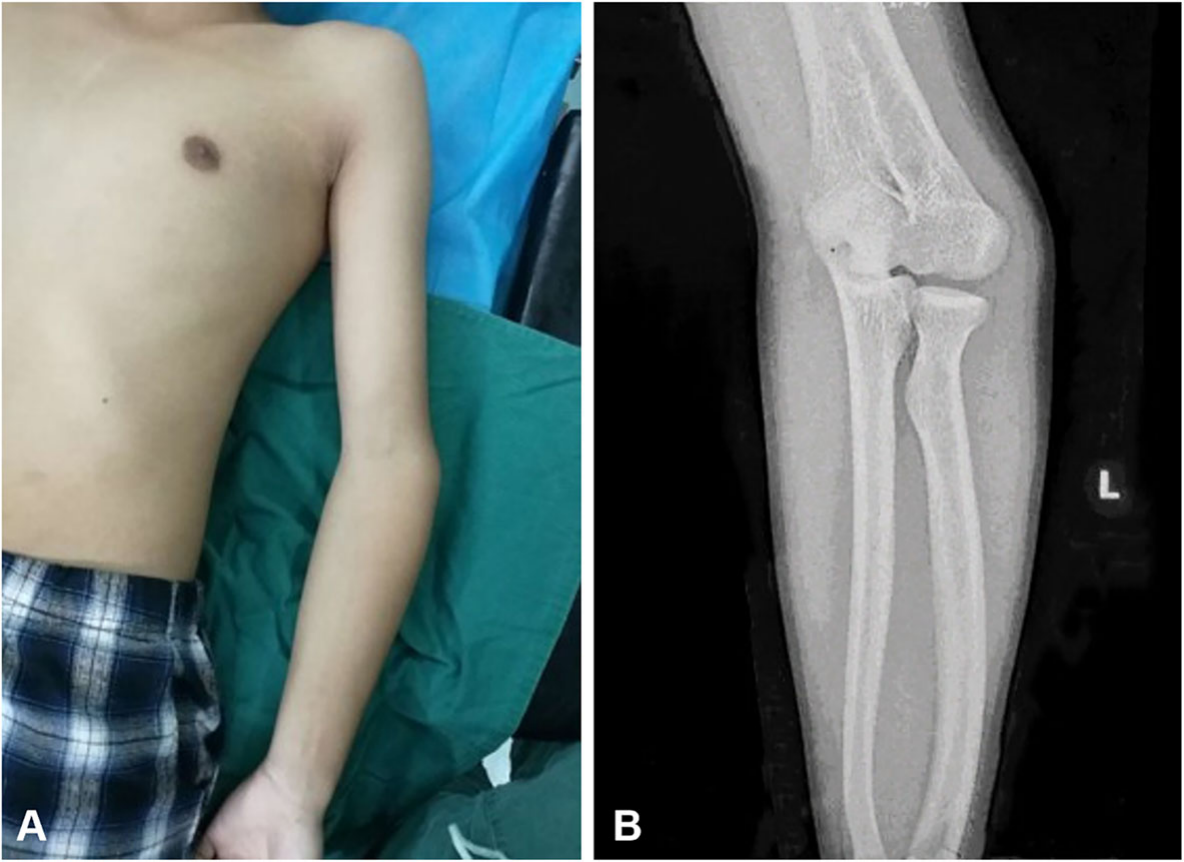
Preoperative appearance and radiographs of a 13-year-old boy with cubitus varus deformity. **a** Preoperative appearance. **b** Preoperative X-rays of the deformed side

**Fig. 5:**
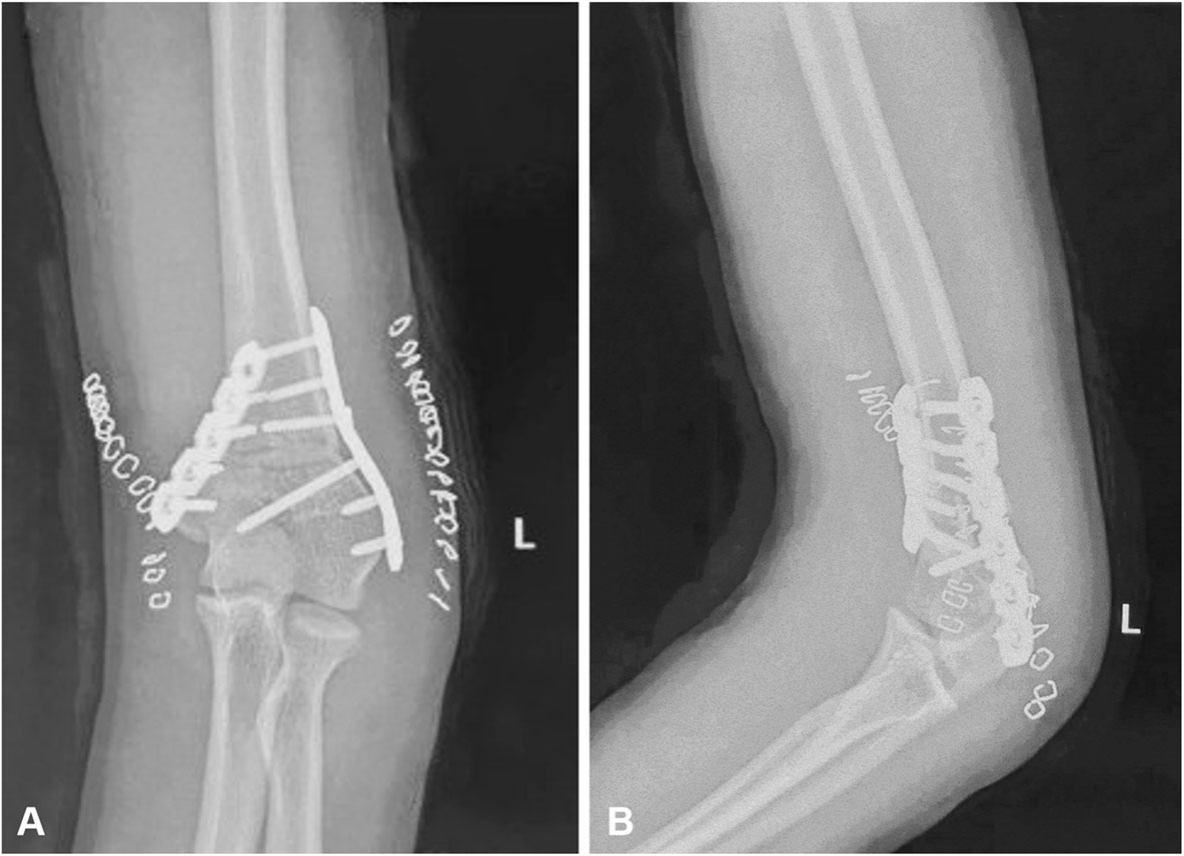
Radiographs after the operation. **a** Positive elbow joint X-ray. **b** Lateral elbow joint X-ray

A male, 13 years old, who experienced a bicycle fall when he was 10 years old and suffered a humeral supracondylar fracture is a typical case. Due to the lack of attention at that time, the cubitus varus deformity appeared 4 months later (Fig. 4). After careful measurements, the cubitus varus angle of his left elbow joint was 15°, the carrying angle of the right elbow joint was 9°, the flexion motion of the left elbow joint was 100°, and the extension motion of the left elbow joint was 10°. After the HSWO assisted by 3D printing osteotomy guide plate, X-ray showed that the osteotomy end was aligned well and the fixation effect was satisfactory (Fig. 5). Three months after the operation, his osteotomy end achieved bone healing. After 18 months of follow-up, the carrying angle of the left side was 8°, the flexion motion of the left elbow joint was 129°, and the extension motion of the left elbow joint was 0°, which was evaluated as excellent according to the Bellemore criteria [16].

## Discussion

Cubitus varus deformity is the most common complication of the distal humeral fractures in adolescents. Although previous studies have confirmed that introversion of distal humeral fracture is the most significant factor for the cubitus varus deformity [17, 18], more and more researchers support that cubitus varus deformity as a three-dimensional deformity in recent years, which includes the varus on the coronal plane, the overextension on the sagittal plane, and the internal rotation on the horizontal plane [4, 19]. Takeyasu et al. [20] also found through the calculations and measurements that 80% of varus cubitus deformities were combined with other dimensional deformities, and only 20% were simply coronal varus deformity. At present, this concept has been recognized by more and more scholars, and the correction of cubitus varus deformity is gradually inclined to the three-dimensional correction [17, 21, 22]. However, it also brings great challenges to the surgeons to a certain extent. It is difficult for the surgeons to accurately adjust the correction angle of each dimension during the operation, which often requires repeated adjustment or only based on the general appearance, and ultimately results in the large deviation and unoptimistic efficacy.

Therefore, we retrospectively compared the efficacy evaluation of the surgery with the surgery assisted by 3D printing osteotomy guide plate in the correction of cubitus varus deformity. In the 3D printing group, the individualized osteotomy guide plate was able to closely attach to the distal humerus, and the one-time osteotomy was successful in all 14 patients. Meanwhile, the process of repeated adjustment was avoided, which enables the surgery to be more convenient, and it is also reasonable to explain that the operation time and intraoperative blood loss in the 3D printing group were significantly less than those in the conventional group. More importantly, the osteotomy guide plate was wedge-shaped as a whole and fitted with Kirschner wire guide holes for fixing the guide plate to prevent the deviation caused by intraoperative slippage. Besides, the compact design of osteotomy guide plate also avoided the expansion of surgical incision. Due to the precise osteotomy, the surgical efficacy was comparable to preoperative expectation, and the 3D printing group also obtained a higher rate of excellent correction than the conventional group (85.8% versus 72.7%, *P* = 0.019). Meanwhile, the satisfaction with postoperative appearance of patients and their parents was greatly improved, and the rate of excellent satisfaction in the 3D printing group was significantly higher than that of the conventional group (92.9% versus 81.8%, *P* = 0.023).

Although the 3D printing guide plate has been widely used in various fields such as the spine, trauma, and arthroplasty currently [14, 23, 24], the applications in adolescent deformity correction have been rarely reported. Previously, Zhang et al. [18] and Jiang et al. [25], respectively, applied the computer navigation templates to correct adolescent cubitus varus deformity and achieved satisfactory short-term effects, but there is still a lack of comparison of the long-term prognosis with conventional surgery group. In this study, there was no significant difference in the total postoperative complication rate between two groups (14.3% versus 18.2%, *P* = 0.371). This result was consistent with that of Yang et al. [26] who applied 3D printing technology to patients with complex elbow fractures, which indicates that 3D printing technology has no obvious advantage in preventing postoperative complications. In addition, at the last follow-up, there was no significant difference in the carrying angle of the deformed side and the elbow joint motions between the two groups. This might be relevant to the fact that the adolescents are still in skeletal development stage and have strong ability of self-regeneration and shaping after the osteotomy. Combined with progressive and planned weight-bearing exercise, both groups of patients showed relatively superior functional results.

Finally, it is still necessary to recognize and point out certain shortcomings in this study. On the one hand, this study is a simple retrospective study with a relatively small sample size. To further evaluate the efficacy of surgery assisted by the osteotomy guide plate, a larger sample size and multi-center prospective studies are still needed. On the other hand, the mean follow-up time of this study was 18 months, and a part of the included adolescent patients was still not at the stage of skeletal maturity. Thus, it is necessary to further improve the follow-up for a longer time. In addition, due to the age difference of adolescent patients, Kirschner wires and reconstruction locking plates were used in this study, and there were certain stability differences among different fixation methods. The control of this parameter should be improved in future studies.

## Conclusion

The application of 3D printing assisted osteotomy guide plate in the correction of adolescent cubitus varus deformity is safe, accurate, and reliable. Compared with the conventional group, the 3D printing group has the advantages of a shorter operation time, less intraoperative blood loss, higher rate of excellent correction, and higher rate of the parents’ excellent satisfaction with appearance after deformity correction. The operation assisted by 3D printing osteotomy guide plate to correct the cubitus varus deformity is feasible and effective, which might be an optional approach to promote the accurate osteotomy and optimize the efficacy.

## Data Availability

The datasets used and/or analyzed during the current study are available from the corresponding author on reasonable request.

## Abbreviations

3D: Three-dimensional
HSWO: Humeral supracondylar wedge osteotomy
CT: Computed tomography
DICOM: Digital imaging and communications in medicine
